# Enhanced Neural Differentiation of Epidermal Neural Crest Stem Cell by Synergistic Effect of Lithium carbonate and Crocin on BDNF and GDNF Expression as Neurotrophic Factors

**DOI:** 10.22037/ijpr.2019.15561.13176

**Published:** 2021

**Authors:** Shirin Ahmadi, Mohammad Nabiuni, Mohammad Tahmaseb, Elaheh Amini

**Affiliations:** a *Department of Cellular and Molecular Biology, Faculty of Biological Sciences, Kharazmi University, Tehran, Iran.*; b *Department of Animal Biology, Faculty of Biological Sciences, Kharazmi University, Tehran, Iran.*

**Keywords:** Neurodegenerative diseases, Epidermal Neural Crest Stem Cell (EPI-NCSCs), Lithium carbonate, Crocin, BDNF, GDNF

## Abstract

Neurodegenerative diseases are incurable and debilitating conditions that result in progressive degeneration of nerve cells. Due to the complexity of conditions in neurodegenerative diseases, combination therapy, including cell and drug therapy is important as a new therapeutic strategy. Epidermal neural crest stem cells (EPI-NCSCs) are among the best choices in cell therapy for various neurological diseases. In this study, the effect of Lithium carbonate and Crocin, considering their effects on cellular signaling pathways and neuroprotective properties were investigated on the expression of neurotrophic factors BDNF and GDNF in EPI-NCSCs. EPI-NCSCs were isolated from the hair follicle and treated with different concentrations of drugs [Lithium, Crocin, and lithium + Crocin] for 72h. Then, trial concentrations were selected by MTT assay. The cells were treated with selected concentrations (Lithium 1 mM, Crocin 1.5 mM, and for co-treatment Lithium 1 mM and Crocin 1 mM) for 7 days. The Real-Time PCR results indicated an increasing in expression of BDNF and GDNF in treated cells as compared with control (^*^p < 0.05, ^**^p < 0.01 and ^***^p < 0.001). The results in this study confirmed and supported the neuroprotective/neurogenesis effects of Lithium and Crocin. It also showed that the proposed protocol could be used to increase EPI-NCSCs differentiation potential into neural cells in cell therapy and combination therapy of neurodegenerative diseases.

## Introduction

Neurons are postmitotic cells in the central nervous system of adult mammalian which largely lose their ability to regenerate after injuries or neurodegenerative disorders. The neural stem cells, located in two regions of the brain, including the subventricular zone (SVZ) and the subgranular zone (SGZ), are not sufficient to repair the disrupted neural circuits under pathological conditions, mainly because of the limited function and distribution of these cells, considered as a major difficulty in treatment of central nerve injuries (1). Spinal Cord Injury (SCI) is one of the famous and widespread types of neurodegenerative diseases with difficulty in treatment. SCI was divided into three phases, including acute, secondary, and chronic phases. After spinal cord injury, a series of events, including loss of neurons, axonal demyelination, and immune and inflammatory responses happened. Current treatment for SCI involves surgery, rehabilitation, cellular, molecular, and combination therapy. The complication of SCI treatment is related to limited neuron regeneration, the presence of free radicals and glutamate excitotoxicity, formation of the glial scar, aggregation of immune cells, and inflammatory responses. Cell therapy can be a useful and effective strategy for this reason and also protect remaining neurons, axons regeneration, and growth factors production. By using a cell replacement strategy (cell therapy), researchers are hoping to create a microenvironment that could allow regeneration and remyelination of axons and the formation of new neurons (2-7).

Recently, adult stem cells that are derived from the neural crest have been considered in cell therapy and due to their presence in the Bulge area of hair follicles are known as Epidermal Neural Crest Stem Cells (EPI-NCSCs) (8, 9). EPI-NCSCs have an ectodermic origin, separated from the neural crest during embryonic development.They can generate all major neural crest derivatives, such as neurons, nerve-supporting cells, melanocytes, and smooth muscle cells. EPI-NCSCs provide a combination of embryonic and adult stem cells benefit (10, 11). These cells exhibit a high level of plasticity and are easily expanded into millions of cells *in vitro*.EPI-NCSCs have also no immunological incompatibility and their isolation method is non-invasive (8).

On the other hand, various studies have revealed several transcription and neurotrophic factors play an important role in the survival, proliferation, and differentiation of stem cells, including neural stem cells. In this regard, neurotrophic factors, especially Brain-derived neurotrophic factor (BDNF) perform a significant role in the evolution and formation of new neurons and synapses (12-14).

EPI-NCSCs, also somehow affect expression and production of various growth factors, such as BDNF, Glial cell-derived neurotrophic factor (GDNF), Nerve growth factor (NGF), and Fibroblast growth factor (FGF) which were required for the survival and regeneration of specific neuronal populations in the embryonic and adult brain (15- 17). 

The complexity of Neurodegenerative disorders reduces the differential ability of EPI-NCSC, on the other hand, it has been accepted that combination therapy can improve the complex condition of neurodegenerative disorders such as SCI (18). In this regard, it is expected that combination therapy using molecular and drug concurrently can promote the process of growth and production of neurotrophic factors or inhibit cell death and inhibitory growth pathways (19). 

Pharmacological therapy is one of the common treatments for SCI. Lithium carbonate is used for bipolar disorders and lithium is a primary and long-term therapy for bipolar disorders, that prevents depression and mania regressive phases (20). The effects of lithium are extensive, including reducing glutamate excitotoxicity, regulation of intracellular calcium concentration, Na + -K + ATPase channel, and releasing neurotransmitters (21)., Lithium can also affect several intracellular signaling enzymes like glycogen synthase kinase-3 (GSK-3) and inositol monophosphatases (IMPAs), which these signaling effects usually result in regulating transcriptional activity through Protein kinase A (PKA) or Cyclic adenosine monophosphate (cAMP) response element-binding (CREB) protein (22, 23).

GSK3 and PKA/cAMP enzymes are CREB regulatory pathways affected by lithium (21, 24, and 25). Lithium’s role in the recovery after SCI is also known by inducing autophagy (26).

Crocin has been studied as a natural product that has fewer side effects compared with chemical drugs. Crocin, a plant-derived carotenoid from *Crocus sativus L*., has been reported to possess anti-inﬂammatory, anti-apoptotic, anti-oxidative, memory improving effects, and selective toxicity against cancer cells (27- 31). 

It is reported that Crocin has protective effects against traumatic brain injury (TBI) in mice, release several pro-inflammatory cytokines, such as tumor necrosis factor-alpha (TNF-α) and Interleukin 1 beta (IL1β), as well as reduction microglial activation and cell apoptosis. Moreover, it was indicated that Crocin promoted the expression of Interleukin 10 (IL-10) after SCI, which has anti-inflammatory activity. This finding proposes that Crocin provides neuroprotective effects by reducing the production of neurotoxic and pro-inflammatory factors from activated microglia (32-35).

In the present study, to find an effective combination therapeutic regimen, the effect of Lithium carbonate, Crocin, and also the synergistic effect of Crocin with Lithium on EPI-NCSCs differentiation is evaluated. 

## Experimental

*Drugs and reagents*

Minimum Essential Medium Eagle Alpha (α-MEM), Fetal Bovine Serum (FBS), Penicillin/Streptomycin, Phosphate buffered saline (PBS), and trypsin were purchased from Bio-Idea (Iran). 3-(4,5-dimethylthiazol-2-yl)-2,5-diphenyl tetrazolium bromide (MTT) was purchased from Sigma (USA), and also Dimethyl sulfoxide (DMSO) and Acetic acid were purchased from Merck (Germany). Lithium carbonate was prepared from Tehran Darou (Iran) and Crocin extracted from *Crocus sativus* was prepared from Puyesh Darou Sina (Iran). A high pure RNA Isolation kit was purchased from Roche (Germany) and a cDNA Synthesize kit was purchased from Pars Tous (Iran). Collagen was extracted from rat tail at Kharazmi University. Chick embryo extract (CEE) (extraction of day-11 chick embryo) was purchased from Shahid Beheshti University, Iran. PCR Thermocycler was Kimiagene (Iran) and Real-time PCR was Corbett (Australia). 

*Animals*

In the present study, Whisker follicles were dissected from 2-week-old Wistar male rats (25-35 g). The Wistar rats were obtained from Kharazmi University, Tehran, Iran. The animals were housed under standard laboratory conditions (temperature of 20 ± 2 °C, relative humidity of 40-45%, and light-dark cycle of 12 h:12 h) and had free access to standard laboratory food and water.

All experimental protocols of this study were approved by the bioethics committee at Kharazmi University in compliance with the standards of the European Communities Council directive (86/609/EEC). Efforts were made to minimize animal suffering and to reduce the number of animals used.

*Collagen extraction*

To explant bulge, the plate was coated with collagen extracted from the rat tail according to the Timpson protocol (36). Briefly, after cutting the rat tail, its tendons were isolated, washed several times with PBS containing 10% Penicillin/Streptomycin, and then put in ethanol 70% for 1 h after removing additional tissues. Then, the tendons were crushed and placed in a solution containing 150 mL deionized water and 170 μL absolute acetic acid and homogenized for 24 h. After homogenization, the solution was centrifuged (Hettich Universal, Germany) and its sediment was separated. Collagen concentration was determined by Nanodrop (Thermo Fisher Scientific, USA) at 280 nm. Eventually, plate was coated with diluted collagen solution (2 mg/mL concentration) based on previous studies (36, 37). 

*Isolation and in vitro expansion of EPI-NCSCs*

To obtain EPI-NCSCs, the whiskers of the 2-week-old male Wistar rat were used. Briefly, the follicles were dissected and cleaned from dermal and adipose tissues. Then, the capsule was cut with a blade and the bulge was rolled out from the capsule and explanted into collagen-coated culture plates (2 mg/mL). At the next step, bulge adhered to the substratum within 1 h and α-MEM supplemented with 10% FBS, 5% CEE, and 1% Penicillin/Streptomycin were added to the plate. After 4-5 days from cell isolation, EPI-NCSCs started to migrate from explant bulges. In Our previous study, characterization of isolated Epidermal Neural Crest Stem Cells (EPI-NCSCs) from bulge explants was confirmed at the gene level using RT-PCR. Briefly, within 2 or 3 days after isolation, cells with stellate morphology emigrated from whisker bulges with increasing numbers over time. The phenotype of migrated cells from bulge explants was confirmed at gene and protein levels with RT-PCR and immunocytochemistry, respectively. After 5 days from the cultivation of explanted bulges in culture medium containing α-MEM with 10% FBS and 5% CEE, the RT-PCR revealed the neural crest stem cell markers such as SOX10, Nestin, GFAP and, β-tubulin ІІІ. After prolonged cultivation of migrated EPI-NCSC in primary media (2 weeks), cells spontaneously differentiated into neural crest progeny, which was confirmed by immunostaining against Nestin, β-tubulin ІІІ, and GFAP, separately. Taken together, these observations validated the expression of pertinent markers and characterized the bulge-derived cells as neural crest-derived cells (37-40).

**Figure 1:**
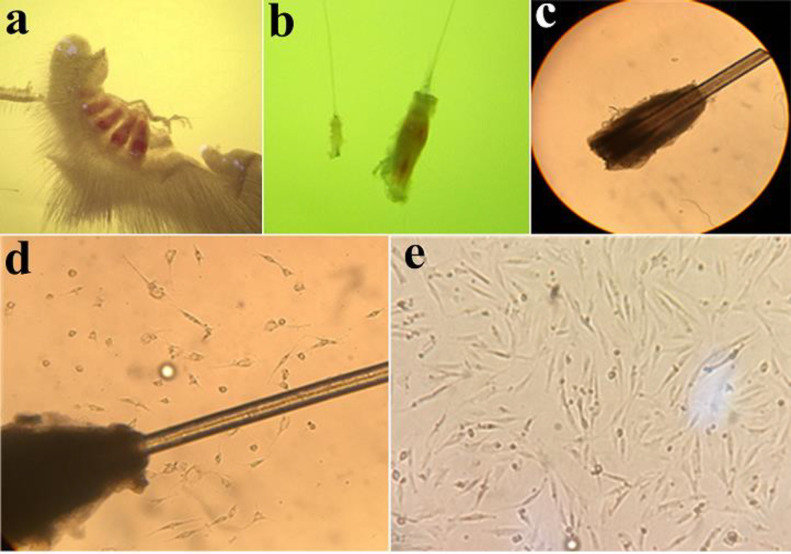
Follicle, Bulge, Bulge explant, and migrated cells. (a) Whisker pad with 4 follicles. (b) A bulge in compression of follicle. (c) Primary bulge explant at the first day of culture (×40). (d) Migration of cells. The bulge appears as dark tissue. It is surrounded by a halo of the cells with stellate morphology that have emigrated from the bulge explant, and which proliferate rapidly under appropriate culture conditions (×100). (e) Migratory cells are present on the collagen substratum. Cells morphology at 5 days after onset of EPI-NCSCs emigration from bulge explants (×100)

In the present study, 4-5 days after observing cell migration, the bulges were removed from the wells to reduce the contamination rate with other later-migrating cell types, such as keratinocytes. In this stage, adherent EPI-NCSCs were dissociated by trypsin and subsequently sub-cultured into a plate (Figures 1a-1e) 

*Evaluation of cell survival by MTT assay*

MTT assay is a common quantitative colorimetric test for the evaluation of cell survival. The alive cells possess mitochondrial dehydrogenase enzymes. This mitochondrial dehydrogenase is active in alive cells and converts 3-(4,5-dimethylthiazol-2-yl)-2,5-diphenyl tetrazolium bromide )MTT) into formazan crystals and determines mitochondrial activity. Since mitochondrial activity is associated with the number of viable cells, this assay can use to obtain cytotoxic effects of various drugs. To obtain an effective and non-toxic concentration of drugs (lithium, Crocin, lithium, and Crocin), EPI-NCSCs were trypsinized and seeded in a 96-wells plate at a density of 5 × 10^3^ cells/per well. After 24 h, the cells treated with α-MEM supplemented with 10% FBS, 1% Pen/Strep and various concentrations of Lithium (0.1, 0.5, 1, 1.2, 1.4, 1.6, 1.8, 2, 4, 8 mM), Crocin (12.5, 50, 100, 200, 500, 1000, 1500, 2000 ,2500 µM) and lithium (1mM) + Crocin (12.5, 50, 100, 500, 1000, 1500, 2000 ,2500 µM) for 72 h. The drug-free α-MEM medium + 10% FBS + 1% Penicillin/Streptomycin wells were used as the control group.

Next, the medium was removed, and 100 µL medium containing 20 µL MTT (5 mg/mL in PBS) was added to each well and incubated for 3-4 h in a CO_2_ incubator. Then, MTT was removed, and 100 µL DMSO was added to each well to dissolve the formazan crystals and absorbance determined at 570 nm by ELISA Reader (Bio-Rad, USA). The results were presented as optical density (OD) (41).

*RNA extraction, cDNA synthesis, and q-RT-PCR*

To evaluate the neurogenic effect of drugs on EPI-NCSCs, the expression of specific neural markers was evaluated. For this purpose, after calculating, the non-toxic concentration of drugs separately (Lithium, Crocin) and concurrently (Lithium and Crocin), EPI-NCSCs cultured at a density of 20000 cells/per well in 6-wells plate was treated with a selected concentration of drugs (Lithium 1 mM, Crocin 1.5 mM, Lithium 1mM and Crocin 1 mM) for 7 days. At the same time, the control group also received no treatment. In this experiment, the medium of both experimental and control groups was renewed on the third and fifth days to prevent toxicity of released substances in the environment and increase drug efficiency. In this study, our purpose was to investigate the effects of selectable drugs on EPI-NCSCs differentiation. Therefore, for evaluating marker genes expression and cell differentiation, drug treatment with those concentrations that don’t have 100% viability was performed. These concentrations inhibit proliferation and let cells proceed differentiation process (1 mM for Lithium carbonate, 1.5 mM, for Crocin, 1 mM for co-treatment).

The concentration neither has a cytotoxic effect on cell survival nor promotes cell proliferation, stimulate cells to have enough time and space for growth and differentiation. Moreover, lithium concentration, which affects proliferation, survival, and differentiation of other stem cells, was confirmed in previous studies (22, 42, and 43).On the seventh day, the medium was removed, the cells were trypsinized, and total RNA was extracted by RNA isolation kit. Then, RNA quantification was carried out using nano-spectrophotometry at 260 and 280 nm and reverse transcribed to cDNA using the easy cDNA synthesize kit with oligo dT primers according to the manufacturer’s protocol. After RNA extraction and cDNA synthesis, Real-time PCR was performed to realize the neurotrophic effect of drugs on the level of GDNF and BDNF mRNA. The sequences of Forward (F) and reverse (R) primers (5^՜^-3^՜^) were as follows (Table 1) (44):

A quantitative real-time PCR was carried out in an Eppendorf Master cycler EP Realplex. Briefly, preliminary PCR was run to optimize the concentration and ratio of each primer set. This assay was accomplished on control and treatment groups. For all the cDNA concentrations, 2 templates were used in a 20 Real-time PCR amplification system of SYBR Green Real Master Mix Kit (Life Technologie, USA) according to the manufacturer’s instruction. Melting curve analysis was performed to confirm the purity of the PCR products. Thermo-cycling conditions were as follows: pre-denaturation at 95 °C for 10 min, and 50 cycles of denaturation at 95 °C for 15 s, annealing at 60 °C for 15 s, and extension at 72 °C for 30 s. The target amplification rate was evaluated from the cycle threshold (Ct) numbers obtained for serial cDNA dilution. The relative expression of mRNA was calculated using the 2^–ΔΔCT^ method with β-actin as a housekeeping gene (45). 

Beta-actin (β-actin) is known as one of the most common housekeeping genes (HKG) and is universally used as endogenous control (reference gene). Also, β-actin was chosen and confirmed as an endogenous control for RT-PCR and western blot analysis of EPI-NCSCs in our previous study (38, 46).

About our gene expression analysis method (2^–ΔΔCT^ method), at first, the LinReg software for estimation of qPCR efficiency was used. The efficiency of qPCR [including of 3 genes (BDNF, GDNF, and beta-actin)] was obtained at 1.95. By application of the Pfaffl method and REST software (Relative Expression Software Tool), the expression of marker genes in control and treatment groups was evaluated. Additionally, the 2^-ΔΔCT^ method (comparative CT method) that was devised by Kenneth Livak and Thomas Schmittgen in 2001 was used to present relative gene expression. Since the qPCR efficiency was 1.95 and near to 2, and on the other hand, the difference between the obtained amount of gene expression from two methods (Pfaffl and 2^-ΔΔCT^) was not significant, the 2^-ΔΔCT^ method was chosen for evaluating and reporting of results. The 2^-ΔΔCT^ method is a simple formula used to calculate the relative fold gene expression of samples when performing real-time polymerase chain reaction; 

Fold change = (2ΔCTtarget (control - sample))/(2ΔCTref (control - sample)) = 2^-ΔΔCT^

The relative expression of BDNF and GDNF was normalized to β-actin using the ΔCT method. Predicted cycle threshold values were exported directly into Excel worksheets for analysis. Relative changes in gene expression were determined by the 2^_ΔΔCT^ method as described previously and reported as the (n-fold) difference relative to the value for a calibrator cDNA (control) prepared in parallel with the experimental cDNAs. Data are presented as the mean ± SEM (47, 48).

*Statistical analysis*

The results are presented as the mean ± SEM from at least three independent experiments. The statistical significance was evaluated by one-way ANOVA and Tukey *post hoc* test. The statistical significance was evaluated using IBM SPSS 22 for Windows software (IBM, Armonk, NY, USA). *p-*value < 0.05 was considered significant.

## Results

*Cell migration*

The culture protocol showed that, after 4-5 days of bulges isolation in collagen-coated plate, the EPI-NCSCs began to migrate from the bulge in the plate surface. After the appearance of cell migration, the bulges were removed from the wells to reduce the rate of contamination with later-migrating cell types (Figures 1d and 1e). 

*Effect of drugs On EPI-NCSCs Viability Using MTT Assay*

**Figure 2:**
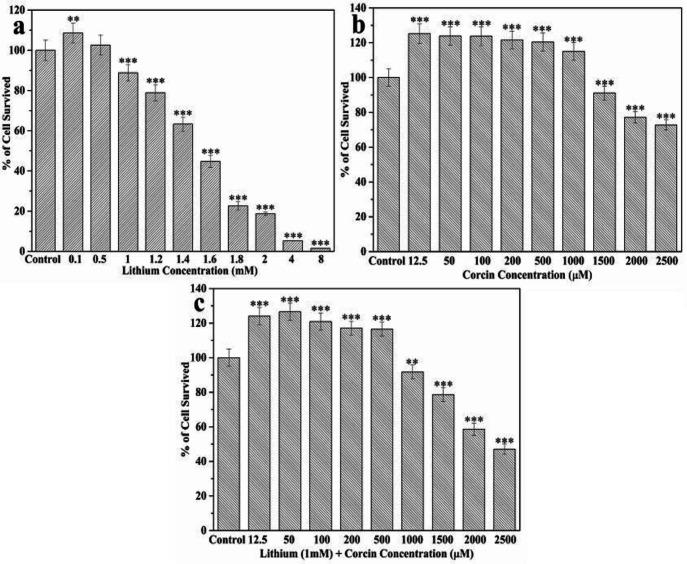
**The effect of Crocin and Lithium in different concentration on EPI-NCSCs viability after 72 h. (a)** **Lithium in concentration 0.1, 0.5, 1, 1.2, 1.4, 1.6, 1.8, 2, 4, 8 mM.** **(b) Crocin in concentration of 12.5, 50, 100, 200, 500, 1000, 2000 and 2500 μM. (c) Co-treatment of Lithium in concentration 1 mM and Crocin in concentration 12.5, 50, 100, 200, 500, 1000, 2000 and 2500 μM.** ^*^ ***p <*** **0.05,** ^**^ ***p <*** **0.01 and** ^***^ ***p <*** **0.001 were considered significant (n = 3, Mean ± SD).**

The purpose of the MTT assay was to find an appropriate concentration of drugs that do not cause cell death and do not significantly increase cell viability. Therefore, the cells were treated with different dosages of lithium and Crocin for 72 h, and cell survival was calculated by MTT assay. The results showed that drugs alternated cell viability in a dose-dependent pattern. According to the purpose of this study, the concentration of 1.5 mM Crocin and 1 mM Lithium was selected to treat EPI-NCSC for 7 days (Figures 2a and 2b). 

Then, for co-treatment of lithium and Crocin, the cells were treated with 1 mM of Lithium and different concentrations of Crocin, which did not increase cell viability and did not show high toxicity (1 mM Lithium and 1 mM Crocin) (Figure 2c).

*Analysis of Gene Expression by Real-Time PCR*

**Figure 3:**
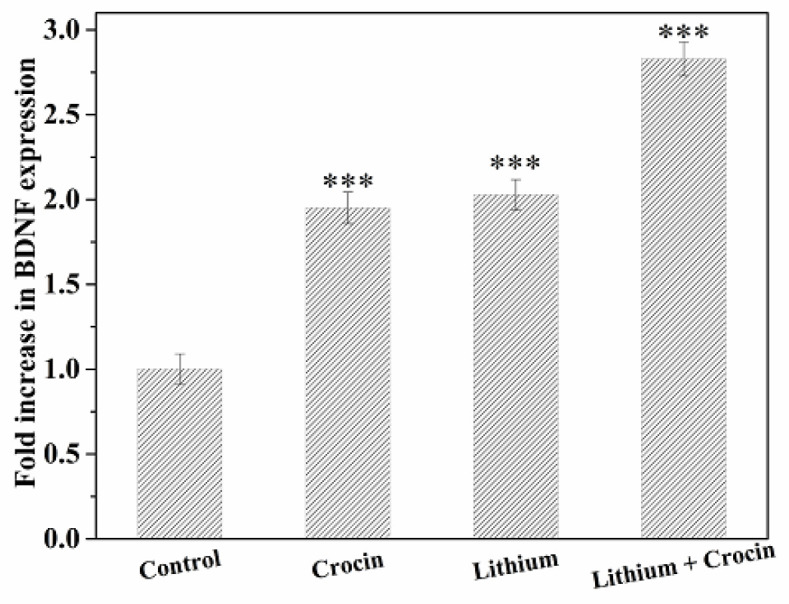
**Evaluation mRNA expression of BDNF in EPI-NCSCs under treatment with Crocin (1.5 mM), Lithium (1 mM), Lithium (1 mM) and Crocin (1 mM). Data were represented as** ^*^ ***p <*** **0.05,** ^**^ ***p <*** **0.01 and** ^***^ ***p <*** **0.001 (method of 2** ^–ΔΔCT^ **).**

**Figure 4:**
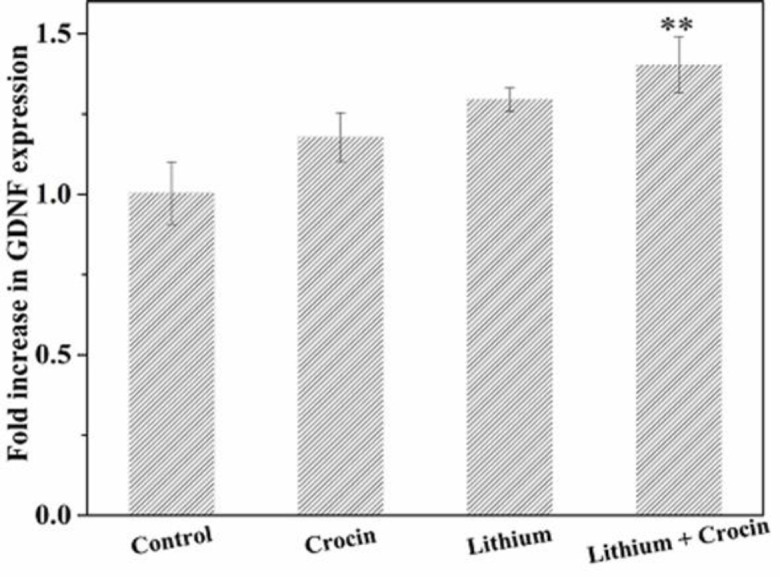
**Evaluation mRNA expression of GDNF in EPI-NCSCs under treatment with Crocin (1.5 mM), Lithium (1 mM), Lithium (1 mM) and Crocin (1 mM). Data were represented as** ^*^ ***p <*** **0.05,** ^**^ ***p <*** **0.01 and** ^***^ ***p <*** **0.001 (method of 2** ^–ΔΔCT^ **)**

To evaluate the neuroprotective/neurogenesis effect of Lithium and Crocin on EPI-NCSCs, the mRNA expression of two selected neurotrophic factors, BDNF and GDNF, was investigated. The cells were treated with differentiating concentrations of Lithium (1 mM), Crocin (1.5 mM), Lithium (1 mM), and Crocin (1 mM). Also, untreated EPI-NCSCs were considered as the control group. The obtained results verified the up-regulation of BDNF and GDNF under treatment with Lithium and Crocin. However, augmentation of GDNF expression was positive but not as more than BDNF expression, which was 1.096, 1.202, and 1.464, respectively (Figures 3 and 4). In addition, this up-regulation was not significant in Lithium and Crocin treatment, separately. 

According to the results, increased expression levels of BDNF and GDNF were more in co-treatment groups (Lithium and Crocin) in comparison with Lithium and Crocin separately, demonstrating the potent neurogenic effect of Lithium and Crocin synergistically on EPI-NCSCs.

**Table 1:** Sequences of different primers used for real-time PCR reaction

**Target**	**Forward**	**Reverse**	**T**_m_
BDNF	5'-CGATTAGGTGGCTTCATAGGAGAC-3'	5'-CAGAACAGAACAGAACAGAACAGG-3'	64
GDNF	5'-GCTGACCAGTGACTCCAATATGC-3'	5'-CCTCTGCGACCTTTCCCTCTG-3'	65
-βactin	5'-TCTATCCTGGCCTCACTGTC-3'	5'-AACGCAGCTCAGTAACAGTCC-3'	64

## Discussion

Complicated conditions of SCI and other neurodegenerative diseases include the limited ability of neuron regeneration, releasing of free radicals and glutamate, the presence of immune cells, inflammatory responses, and formation of glial scar cause the current therapies to be far from effective treatments. Thus, neuron regeneration, protecting remaining cells, and balancing the microenvironment in the lesion region are important goals in the studies of neurodegenerative diseases (1, 18). 

Appropriate and successful treatment includes treatment with protective agents, protection of residual cells, increment of growth factors, and enhancement of axonal regeneration. The present study was conducted to verify whether Lithium carbonate and Crocin affected neurotrophic factor expression in EPI-NCSCs. The principle objective of this study was evaluation the impact of Lithium and Crocin on survival, viability, and expression of selective neurotrophic factors in Epidermal Neural Crest Stem Cells.

In 2014 Nuna A. Silva *et al.*, reported that new therapies, including cell therapy, molecular therapy, and combination therapy, can be effective treatments (18). Moreover, the effectiveness of cell therapy and stem cell transplantation for spinal cord injury was proven (7).

Numerous studies have shown EPI-NCSCs can be an effective candidate for cell transplantation and cell therapy. Furthermore, the role of neurotrophic factors such as BDNF and GDNF has been verified in cell survival, neuron regeneration, and neural stem cell differentiation into neuronal cells (12). Neurotrophic factors activated the Ras-dependent signaling pathway, which caused neurogenesis, neuron growth, and regeneration (15).

In view, it could be proposed that the increased expression of BDNF and GDNF can protect EPI-NCSCs and enhance their ability for differentiation into neural and neuronal cells, and results to improve the success of therapeutic transplantation in combination therapy based on EPI-NCSCs in neurodegenerative diseases.

The obtained results demonstrated that *in-vitro* treated EPI-NCSCs with Lithium carbonate, Crocin, and co-treatment with selective concentration induce expression of BDNF and GDNF compared with non-treated EPI-NCSCs. This led us to develop a novel mechanism to predict the neuronal fate of EPI-NCSCs before transplantation to enhance therapeutic efficiency.

Lithium is well known for its role as a direct and indirect inhibitor of glycogen synthase kinase-3 (GSK-3), a component of the Wnt signaling pathway. The Wnt signaling pathway is a critical pathway for a variety of cellular processes, including cell proliferation, neurogenesis, and synaptogenesis in embryonic and adults nervous systems (24, 42). GSK3 is one of the Lithium goals and enzymes in CREB regulatory pathways. CREB acts as a transcription factor, and has a positive effect on the expression of the BDNF (a neurotrophic factor) and BCL-2 (an anti-apoptotic) and also reduces the expression of p53 and Bax (BCL-2-associated X) as pro-apoptotic factors (21, 24). With consideration that the CREB/BDNF, BCL2 pathway is involved in observed effects of Lithium carbonate including neuroprotective, neurogenesis, and cell survival. Bcl-2 plays a key role in promoting the regeneration of injured axons in the CNS. These findings elucidated that Lithium can enhance axonal regeneration through a Bcl-2–dependent mechanism. Lithium affects the proliferation and viability of many types of stem cells, such as neural stem cells, mesenchymal stem cells, hematopoietic stem cells, and embryonic stem cells. Further, Lithium influences the differentiation of neural progenitor cell, mesenchymal stem cells and increase newly formed neurons in the spinal cord to a specific phenotype (43, 50 and 51). 

The obtained results of the present study confirmed the previous results and showed that Lithium increased cell viability in a dose-dependent way and improved the BDNF and GDNF expression. However, the amount of increase for BDNF was more than GDNF. Moreover, this augmentation at the expression in co-treatment of Lithium and Crocin was higher than each drug, separately (Lithium or Crocin).

Based on previous studies, it could be proposed that inhibition of GSK-3 in EPI-NCSCs may facilitate CREB activation and increase the expression of BDNF and GDNF, consequently, enhance differentiation of these cells into neural cells.

Related to the involved signaling pathway, lithium influences can be direct through competition with magnesium (Mg^2+^) that acts as a cofactor for some enzymes such as IMPase and GSK3, or indirect through its products such as BDNF, which affects Akt enzyme and ERK pathway (Ras pathway) (52). 

On the other hand, the previous ﬁndings have shown the saffron has effects on the inhibition of inﬂammation pathways. Crocin as a neuroprotective agent can act as an antioxidant agent, which decreases neuronal cell death by increment glutathione level and inhibiting the c-Jun N-terminal kinase (JNK) pathway. Recent studies have also shown Crocin exerts neuroprotective effects through attenuation of the neurotoxic molecular level (53, 54).

Regarding the anti-inﬂammatory effects of Crocin, Firouzi *et al*. reported that pretreatment of Crocin induces neuroprotective effects on traumatic brain damage, decreased microglial activation, apoptosis, and pro inﬂammatory cytokine release through activation Notch signaling pathway (54).

The results indicated that the expression of BDNF and GDNF in EPI-NCSCs was increased under treatment with Crocin. Significantly, the increase in levels of these neurotrophic factors was more for co-treatment of Crocin and Lithium. Although Crocin has not solely considerable neurotrophic and neuroprotective effects, when it is used with Lithium, the neurotrophic effects of this drug (Lithium carbonate) could be increased. This effect can be related to the anti-apoptotic effects of Crocin. Previous studies revealed that Crocin inhibits neuronal apoptosis by suppression of caspase 3. Also, the anti-edema effect of Crocin is probably associated with its anti-apoptotic and anti-inflammatory effects (56).

Cell therapy based on EPI-NCSCs has a lot of potential for therapy, including replacement of glial and neuronal cells, induction of angiogenesis, and secretion of neurotrophic factors (57). 

In the present study, the obtained results were consistent with the expected results based on previous studies. These findings can point to the role of Crocin as a complementary drug in SCI and neurodegenerative disease treatment.

There was one point about GDNF expression, relative and inconsiderable increment in GDNF expression in comparison with BDNF in all samples, which can be due to greater effects of these drugs on enzymes and signaling pathways that leading to BDNF expression, like the Ras-Raf-MEK-ERK pathway.

## Conclusion

In this study, isolated EPI-NCSCs were treated with different concentration of drugs (Lithium carbonate, Crocin, and co-treatment) and then, BDNF and GDNF expression was evaluated. It was suggested that although Lithium carbonate and Crocin may not be sufficient to enhance generated newly neurons, they can improve cell survival, reduce the death of remaining cells through activation a lot of cell-survival factors including neurotrophic/neuroprotective such as BDNF, increase neuronal differentiation by activating of the Wnt/MAPK, ERK-, and CREB-dependent signaling pathways by enhancing neurotrophic factors expression like BDNF and GDNF in the region of the lesion.
